# Protein–Lipid Interactions: From Molecular Recognition to Cellular Organization and Disease

**DOI:** 10.3390/membranes16060193

**Published:** 2026-06-03

**Authors:** Nikolas Nikolaidis

**Affiliations:** Department of Biological Science, Center for Applied Biotechnology Studies, Titan Supercomputing Center, Director, Project EAGER–Genomics Education and Research, California State University Fullerton, Fullerton, CA 92831, USA; nnikolaidis@fullerton.edu

Cell biology has traditionally relied on a useful working division of labor: proteins are the primary agents of function, and lipids provide the structural context in which that function unfolds [1,2]. While this framing has been enormously productive, the field has long recognized that it is incomplete. What the studies in this Special Issue demonstrate collectively is how far the implications of that incompleteness extend. They reinforce the growing consensus that lipids are not simply the background of cellular function. Instead, they are active determinants of its logic, governing protein localization, conformation, and activity, and, in many cases, defining pathological outcomes.

Protein–lipid interactions regulate subcellular localization, modulate protein conformation and activity, and define the organizational principles of cellular membranes. These are not peripheral phenomena [1,2,3,4,5]. They are, as the studies collected here reveal, central to how cells encode spatial and biochemical information—and to how that encoding breaks down in disease. The goal of this editorial is not to summarize what each contribution found, but to trace the conceptual thread that runs through all of them, and to articulate what the field still needs.

**Lipid Identity Is Read, Not Ignored**

The first principle that emerges from this collection is one of specificity. Lipids are not interchangeable, and proteins do not engage membranes indiscriminately. Two studies in this issue make this argument from very different biological systems and reach strikingly convergent conclusions.

Melnikova and colleagues examined the interaction between a lentil lipid transfer protein, Lc-LTP2, and phosphatidylinositol (4,5)-bisphosphate—a low-abundance but highly bioactive phosphoinositide. Using a combination of overlay assays, molecular modeling, and liposome experiments with targeted mutants, they showed that PI(4,5)P_2_ binds at a defined entrance to the protein’s hydrophobic cavity, with two conserved residues—Arg45 and Tyr80—playing distinct and essential roles. Arg45 governs initial contact and retention; Tyr80 mediates membrane docking. This is not generic lipid affinity. It is molecular recognition with the precision normally associated with protein–protein or protein–nucleic acid interactions. The authors propose that this interaction links LTP function to plant signal transduction—a conclusion with implications well beyond the plant kingdom, given PI(4,5)P_2_’s broad signaling role across eukaryotes.

Rudajev and Novotny approached the same principle from a neurobiological perspective, examining how ganglioside GM1—concentrated in lipid-raft microdomains of neuronal membranes—shapes the conformational behavior of amyloid-β. GM1 does not simply co-localize with Aβ; it actively promotes specific conformational transitions that accelerate aggregation and enhance toxicity toward the very membranes in which it resides. Lipid composition, in this case, is not an incidental feature of the environment—it is a determinant of pathological outcome. The identity of the lipid matters, the spatial organization of that lipid matters, and the protein cannot be understood without it.

**Membranes Are Not Uniform Surfaces**

If specific lipid species carry discrete molecular information, the physical properties of the membrane—its geometry, phase behavior, and compositional heterogeneity—impose an additional layer of constraint on protein behavior. Two structural studies in this collection illustrate how deeply membrane architecture shapes protein function.

Park and colleagues characterized the N-terminal region of caveolin 3 via solution NMR in a detergent micelle environment, revealing a structurally complex domain with internal communications between the flexible N-terminus, a pH-sensitive helical region, a signature motif, and the scaffolding domain. These are not independent elements; they engage in dynamic crosstalk that is sensitive to environmental conditions. Several disease-linked mutations in Cav3 map precisely to this region, suggesting that the structural integrity of the membrane-facing N-terminus is not merely a folding requirement; it is a functional necessity. Caveolae, the membrane invaginations organized by caveolins, are defined by their lipid environment, particularly their cholesterol and sphingolipid composition. The protein cannot be separated from that context without losing explanatory power.

Titus and colleagues offered a complementary perspective by examining perilipin 3 at the lipid droplet interface—an organelle whose surface is a phospholipid monolayer rather than a bilayer. This geometric distinction has consequences. The full-length protein and its C-terminal domain show different affinities and insertion behaviors at the monolayer interface compared to the aqueous surface, with the C-terminus displaying a specific preference for phosphatidylethanolamine-containing monolayers that the full-length protein does not share. The two domains of perilipin 3 thus make distinct contributions to lipid droplet targeting, and both contributions depend on the physical chemistry of the interface. Membranes, these studies collectively argue, are not uniform surfaces awaiting the arrival of proteins. They are structured environments that proteins have evolved to read.

**Membranes as Programmable Platforms**

The recognition that protein–lipid interactions follow defined rules carries a corollary: those rules can, in principle, be exploited. Zaruba and colleagues demonstrated this explicitly, engineering the surface of bacterial outer membrane vesicles with GPI-anchored eukaryotic proteins. GPI-anchored proteins, whose lipid anchor inserts into the outer leaflet of eukaryotic membranes, can be transferred to OMV surfaces through a process the authors describe as molecular painting. The result is a prokaryotic vesicle displaying eukaryotic proteins with authentic folding and post-translational modifications—a hybrid construct with potential applications in vaccine design, gene therapy, and diagnostics. If these interactions are programmable in engineered systems, their dysregulation in native cellular contexts is likely to be equally consequential.

Beyond its biotechnological significance, this study makes a conceptual point. The chemistry governing GPI anchor insertion is sufficiently conserved to function across the evolutionary distance between bacteria and mammals. The rules of protein–lipid interaction are not idiosyncratic to any one system; they reflect deep physical and biochemical logic. This portability is precisely what makes understanding those rules so consequential.

**What We Cannot Yet Measure—and What That Costs Us**

Understanding protein–lipid interactions in their native context requires tools that the field does not yet fully possess. Most mechanistic knowledge in this area has been derived from reconstituted systems: purified proteins, model membranes, and detergent micelles. These systems are tractable and informative, but they are, inevitably, simplifications.

Jing and colleagues addressed this limitation directly by integrating surface plasmon resonance imaging with cell edge deformation tracking to quantify protein binding kinetics in whole cells at single-cell resolution. By quantifying protein binding kinetics directly on the basal membrane of living cells, this approach begins to move the field beyond the inherent simplifications of model bilayers. However, it serves primarily as a methodological bridge, offering a more realistic physical context while highlighting that the deeper questions of protein orientation and sub-molecular stability remain just out of reach. This is a meaningful advance. It also highlights, by contrast, what remains inaccessible: the orientation and conformational state of proteins upon membrane engagement, the stability and lifetime of specific contacts, the distinction between lipid-specific and promiscuous binding, and the coupling between membrane engagement and changes in protein function—all of which remain difficult to resolve in live cells under physiological conditions. The consequences of this gap are not theoretical; they are already visible in disease.

Two studies in this collection document what happens when protein–lipid interactions are dysregulated across time or under pathological conditions. Timsina and Mainali reviewed the progressive association of α-crystallin—a small heat shock protein essential for lens transparency—with the fiber cell plasma membrane during aging and cataract formation. The cytoplasmic pool of α-crystallin, responsible for suppressing protein aggregation, diminishes as membrane-bound α-crystallin accumulates. Proteins that function as chaperones in the cytoplasm become sequestered at the membrane, coinciding with the onset of aggregation.

The molecular basis of this redistribution—whether it reflects specific lipid headgroup recognition, changes in membrane physical state, or stress-induced conformational changes in the protein itself or the surrounding lipid environment—remains unresolved. Similarly, the role of GM1 in directing amyloid-β toward toxic conformations represents a case where a normal membrane component, present in neuronal rafts throughout life, becomes a pathological catalyst under conditions that remain incompletely characterized. In both cases—cataract and neurodegeneration—the disease is not caused by a broken protein in isolation, but by a disruption of the relationship between a protein and its membrane.

**What Remains**

The seven studies assembled here span kingdoms, organelles, methodological approaches, and disease contexts. They do not constitute a unified theory of protein–lipid interaction. They constitute something more honest: a set of converging observations that make visible the need for such a theory.

A genuine mechanistic framework will require integrating structural resolution with lipidomic depth, quantitative biophysics with live-cell dynamics, and molecular detail with systems-level context [1]. Key parameters—orientation, stability, lipid specificity, functional coupling—must become measurable in native environments, not just in reconstituted ones. Moreover, the connection between molecular events at the membrane and pathological outcomes at the cellular and organismal levels must be drawn with mechanistic precision, not correlation [1,2,3,4,5].

This Special Issue does not resolve that challenge. It maps its contours—and, in doing so, argues that protein–lipid interactions are not a peripheral aspect of cell biology, but one of its defining organizational principles.
